# Influence of seasonal changes and salinity on spinach phyllosphere bacterial functional assemblage

**DOI:** 10.1371/journal.pone.0252242

**Published:** 2021-06-01

**Authors:** Abasiofiok M. Ibekwe, Selda Ors, Jorge F. S. Ferreira, Xuan Liu, Donald L. Suarez

**Affiliations:** 1 US Salinity Laboratory, USDA-ARS, Riverside, CA, United States of America; 2 Ataturk University, Department of Agricultural Structures and Irrigation, Erzurum, Turkey; Assam Agricultural University Faculty of Agriculture, INDIA

## Abstract

The phyllosphere is the aerial part of plants that is exposed to different environmental conditions and is also known to harbor a wide variety of bacteria including both plant and human pathogens. However, studies on phyllosphere bacterial communities have focused on bacterial composition at different stages of plant growth without correlating their functional capabilities to bacterial communities. In this study, we examined the seasonal effects and temporal variabilities driving bacterial community composition and function in spinach phyllosphere due to increasing salinity and season and estimated the functional capacity of bacterial community16S V4 rRNA gene profiles by indirectly inferring the abundance of functional genes based on metagenomics inference tool Piphillin. The experimental design involved three sets of spinach (*Spinacia oleracea* L., cv. Racoon) grown with saline water during different seasons. Total bacteria DNA from leaf surfaces were sequenced using MiSeq® Illumina platform. About 66.35% of bacteria detected in the phyllosphere were dominated by four phyla- *Proteobacteria*, *Firmicutes*, *Bacteroidetes*, and *Actinobacteria*. Permutational analysis of variance (PERMANOVA) showed that phyllosphere microbiomes were significantly (*P* < 0.003) affected by season, but not salinity (*P* = 0.501). The most abundant inferred functional pathways in leaf samples were the amino acids biosynthesis, ABC transporters, ribosome, aminoacyl-tRNA biosynthesis, two-component system, carbon metabolism, purine metabolism, and pyrimidine metabolism. The photosynthesis antenna proteins pathway was significantly enriched in June leaf samples, when compared to March and May. Several genes related to toxin co-regulated pilus biosynthesis proteins were also significantly enriched in June leaf samples, when compared to March and May leaf samples. Therefore, planting and harvesting times must be considered during leafy green production due to the influence of seasons in growth and proliferation of phyllosphere microbial communities.

## 1. Introduction

The phyllosphere is the aerial part of plants and the most prevalent bacterial habitats on plants, as well as the primary center of photosynthetic activities. For leafy greens like spinach, it is the edible portion of the herbaceous plant with direct impact on human health due to the presence of potential human pathogens [1]. Many culture-independent studies have shown that the phyllosphere harbors many hundreds of unique bacterial taxa with bacterial community composition varying across plant species and more diverse than previously thought using culture-dependent methods [1–4]. It has been shown that phyllosphere microbiomes are related to specific processes at the interface between plants, microorganisms and the atmosphere [5]. These authors noted that the phyllosphere is a major bacterial habitat on earth and these microbes are exposed to extreme, stressful, and changing environments. Also, the phyllosphere microbiota and the atmosphere present a dynamic continuum of interactions with volatile organic compounds and atmospheric trace gasses. Due to these interactions, bacterial communities on the phyllosphere are subjected to large changes in temperature, soil water content, UV radiation, relative humidity, and leaf wetness [6–8]. These changes combined with changes in soil physiochemical factors may result in stressful conditions for the growth of leafy green vegetables such as spinach. These environmental changes may influence the ability of human pathogens in association with other bacterial community members to persist in the phyllosphere [9], and the overall changes temperature, soil water content, UV radiation, and relative humidity can elevate the problems of salinity in soil [10].

Impacts of salinity on plants and microbes are generally through selective ion toxicity [11,12]. Changes in soil salinity may be one of the parameters impacting the use of recycled wastewater for agricultural irrigation [13]. In the next few years, the semiarid region of southwestern USA would likely experience increases in temperature, lesser precipitation, and more severe droughts [14,15]. Our understanding of leaf microbiota as providers of specific functions, for example, pathogen exclusion [16] and nitrogen fixation [17], relies on continued efforts to catalog the bacterial communities on plant foliage. Data analysis involving changes in environmental parameters influencing microbiome community changes often use the 16S rRNA gene sequencing methods while functional information on these changes requires analysis based on shot gun sequencing [18]. However, 16S rRNA data may be used to predict the functional attributes of bacterial assemblages based on new bioinformatic tools such as PICRUSt [19], Tax4Fun [20], and Piphillin [21]. The main objectives of this study were to determine bacterial composition at different seasons, and to examine functional capabilities of bacterial communities on the phyllosphere induced by seasonal changes and salinity.

## 2. Material and methods

### 2.1. Experimental treatments

These studies were conducted outside in closed recirculating tanks [22] filled with a mixture of loamy sand and peat moss (here fore designated as sand tanks). The system consists of 24 experimental plant growth units at the U. S. Salinity Laboratory in Riverside, CA. The experiments were conducted to determine the interaction of salinity and drought treatments on phyllosphere community composition. Experiments 1 started on 7 December 2012 and end in March 2013, and experiment 2 started on 14 March, 2013 and ended in May 2013 in large sand tanks as previously described [22]. Seeds were seeded and seedlings thinned to 25 plants per row in sand culture tanks as previously discussed [23] (S1 Fig). Experiment 3 was conducted in smaller sand tanks as previously described [24] and started in late April 2013 and ended in June. All tanks were irrigated with modified half-strength Hoagland’s nutrient solution combined with various salinity levels. Starting nutrient solution was made up in Riverside tap water to flush the system. The three experiments utilized modified half Hoagland’s solution with (in mM): 2.5 Ca (NO_3_)_2_, 3.0 KNO_3_, 0.17 KH_2_PO_4_, 1.5 MgSO_4_, 0.05 Fe as sodium ferric diethylenetriamine pentaacetate (NaFe-EDTA), 0.023 H_3_BO_3_, 0.005 MnSO_4_, 0.0004 ZnSO_4_, 0.0002 CuSO_4_, and 0.0001 H_3_MoO_4_. The irrigation waters were targeted at EC_i_ of 4, 7, 9, 12, 15 dS m^−1^ by adding CaCl_2_, MgCl_2_, NaCl, Na_2_SO_4_ to the base tap water-nutrient solution, and the salt concentrations used were based on EXTRACT Chem model [25] to predict the ion composition needed to achieve the target EC values. The control solution was without the added salinity nutrient and was maintained at an EC of 0.85 dS m^−1^. Salinity treatments started after the first pair of true leaves was fully expanded on all the plants as previously reported [23] as well as tank irrigation. The three experiments were randomized design with three replications [22,23] including control with an EC at 0.85 dS m^−1^ and two different water types dominated by sulfate and chloride ion during the first experiment, while the second and third experiments only chloride ion water type. In these subsequent experiments we used only chloride water type since the first experiment did not show any statistical differences in spinach yields at any salinity levels between sulfate and chloride water types [22]. The concentrations of Na, K, Mg, Ca, and total- S [22] on the phyllosphere samples were as previously determined [22]. The average temperatures (°C) and reference evapotranspiration (ET_0_) that occurred during the experiment was acquired from the California Irrigation Management Systems (CIMIS) weather station no. 44 at University of California Riverside (S2 Fig), California [26]. About five leaf samples from one plant in the middle of the plot were cut above the soil surface with sterile blade and placed in stomacher bags and weighed. A total of five plants were sampled per plot, and the DNA from these plants were pooled into one tube per replicate for sequencing. A total of three replicates were used for each treatment. Total bacterial community DNA was recovered from the plant material by homogenization with 100 ml of PBS for 2 min at 260 rpm in a Seward Stomacher 400 Circulator (Seward Ltd., London, UK). Concentrated samples were used for isolation of the genomic DNA used for MiSeq sequencing.

### 2.2. DNA extraction and V4 16S Illumina MiSeq sequencing

Phyllosphere DNA samples were extracted with the Power soil DNA Kit (MoBio Laboratories, Solana Beach, CA) from triplicate plots and stored at –20°C after further cleanup steps with DNA Clean and Concentrator (Zymo Research Corp- Irvine CA). DNA was quantified using a Nanodrop ND-2000 C spectrophotometer (Nanodrop Technologies, Wilmington DE) and Qubit HS kit (Fisher Scientific) and run on 1.0% agarose gel. DNA samples were sent to Second Genome (San Bruno, CA, USA) for V4 16S Illumina MiSeq sequencing. The V4 region of the 16S rRNA genes was amplified be Second Genome using fusion primers 515F (5′-GTGCCAGCMGCCGCGGTAA-3′) and 806R (5′-GGACTACVSGGGTATCTAAT-3′) [27], for sequencing using the MiSeq (Illumina, San Diego, CA, USA) instrument. Due to the presence of plant chloroplast DNA in surficial vegetable microbial metagenomes that may be confused with bacteria DNA, plant-derived chloroplast sequences were downloaded from Greengenes (May 2013 release) and removed before sequence analysis, and the sequence were rarefied to a standard number based on the lowest sequenced size [28] (Guron et al., 2019). Sequence data are available on this link to the general public: https://ars.usda.gov/ARSUSERFILES/20361500/PUBLIC%20DATA/DNA-SEQUENCE-OTU.DOCX.

### 2.3. Statistical analysis of sequence data

Statistical analysis was done using QIIME [29], UCLUST [30] and MOTHUR [31], and sequences clustered into reference OTUs taxonomic classification using the Greengenes database [32]. Sequenced paired-end reads were processed using USEARCH [33], and clustered at 97% similarity by UPARSE (*de novo* OTU clustering). Clustering, chimera filtering, quality filtering was done as previously discussed [23]. Alpha-diversity was calculated to estimate sample richness and Shannon diversity and Beta-diversity metrics was calculated for the inter-comparison in a pair-wise fashion to determine dissimilarity score and store it in a distance dissimilarity matrix. Abundance-weighted sample pair-wise differences were calculated using the Bray-Curtis dissimilarity. All analyses were generated using Second Genome R package (vegan: R package version 2.2–1) as previously reported [23]. Univariate differential abundance of OTUs was tested using negative binomial noise model for the over dispersion and Poisson process intrinsic to this data, as implemented in the DESeq2 package [34], and as described for microbiome applications [35].

### 2.4. Inference of metagenomes

Piphillin was used to leverage the most up-to-date genome database for metagenome prediction from 16S rRNA sequence data [21], using the Kyoto Encyclopedia of Genes and Genomes (KEGG) (KEGG 70.1 database). A genome was inferred for each 16S rRNA OTU based on the sequence identity between an OTU’s representative sequence and the nearest neighbor 16S rRNA sequence from the genome databases restricted to a minimum identity of 97%. OTU abundance was normalized by 16S rRNA copy numbers and then multiplied by the gene contents of each inferred genome to predict each sample’s metagenome.

### 2.5. KEGG Pathways and genes significance testing

To identify differentially abundant pathways and genes, a Wilcoxon Rank Sum test was employed. Where samples could be paired across categories, a paired Wilcoxon Signed Rank test was employed. P-values were adjusted by Benjamini-Hochberg procedure to control for false discovery rates from multiple testing.

## 3. Result and discussion

Alpha diversity index (Shannon index of diversity) was significantly higher in June (Table 1). Our study agrees with a study of leaf age and seasonality of the tree species *Quercus ilex* in the Mediterranean forest which revealed an increase in the richness of epiphytic bacteria with increasing time of colonization in summer [36]. Other studies have suggested that the bacterial community composition in leafy greens change with season and not solely with leaf maturation [9]. Our study is one of the few studies that show seasonal effects of phyllosphere bacterial community changes on leafy greens and confirmed the findings of Williams et al. [9], where, for the first time, a seasonal effect was reported. The study by Williams et al. [9] was conducted in the Salinas Valley, CA, USA with Romaine lettuce and two irrigation regimes, whereas this study was conducted in southern California with drip irrigation and three seasons. Most of the leafy green productions in Salinas Valley are grown from June to September, whereas leafy green production in southern California, USA is generally grown during the winter months from November to March. Therefore, it is expected that bacteria will respond differently to the temperature differences.

**Table 1:** Mean (SD) values for alpha diversity metrics for each study group.

Month	Type	OTUs	Shannon (H’)
**June**	leaf	3334 (468)	2.89 (1.22)
**March**	leaf	755 (243)	0.977 (0.42)
**May**	leaf	1557 (258)	1.71 (0.291)

Bacteria were relatively dominated by four phyla- *Proteobacteria*, *Firmicutes*, *Bacteroidetes*, and *Actinobacteria* -accounted for about 66.35% of taxa detected in the phyllosphere (Table 2). Three bacterial taxa at the family level were relatively dominant in the phyllosphere (*Enterobacteriaceae* (3.38–14.51%), *Halomonadaceae* (0.0174–3.12%), and *Pseudomonadaceae* (0.49–11.5%), with other taxas registering less than 1% (Table 2).

**Table 2:** Most abundant taxa at the phylum and family levels.

Phylum	leaf	Family	leaf
*Proteobacteria*	33.9 (9.7)	*Enterobacteriaceae*	12 (10)
*Firmicutes*	27.8 (5.5)	*Flavobacteriaceae*	0.276 (0.447)
*Bacteroidetes*	2.39 (1.92)	*Comamonadaceae*	0.574 (0.53)
*Actinobacteria*	2.26 (2.5)	*Pseudomonadaceae*	4.81 (6.09)
*Chloroflexi*	0.812 (1.28)	*Sphingomonadaceae*	0.685 (0.834)
*Planctomycetes*	0.733 (1.09)	*Halomonadaceae*	1.1 (2.34)
*Acidobacteria*	0.699 (0.98)	*Cytophagaceae*	0.433 (0.436)
*Verrucomicrobia*	0.209 (0.32)		

### 3.1. Comparison of leaf microbiomes in response to salinity

**Fig 1:**
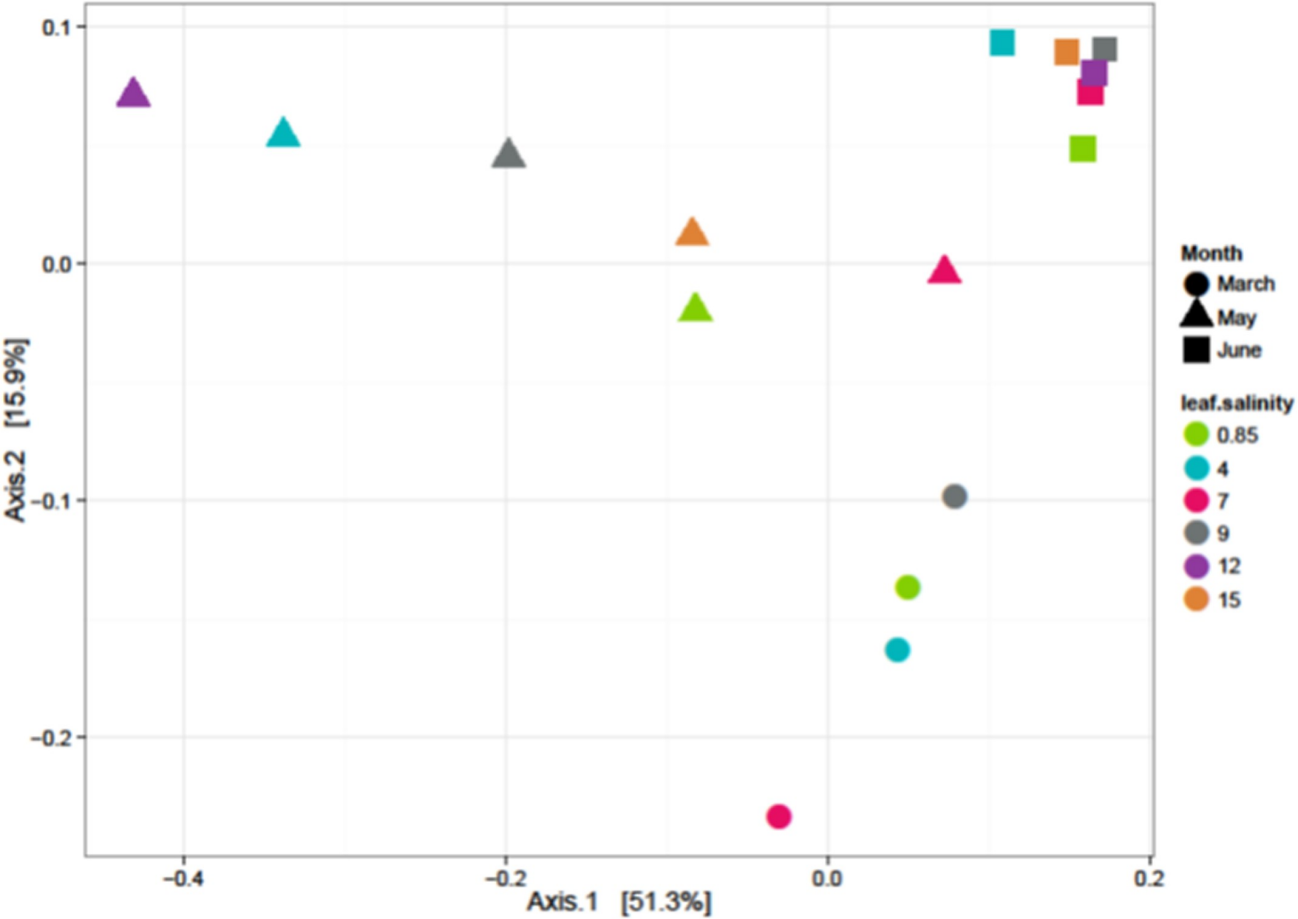
Weighted ordination in the phyllosphere: Dimensional reduction of the Bray-Curtis distance between microbiome samples, using the PCoA ordination method.

Salinity did not significantly influence bacterial beta-diversity on the phyllosphere using weighted abundances (Fig 1).

**Fig 2:**
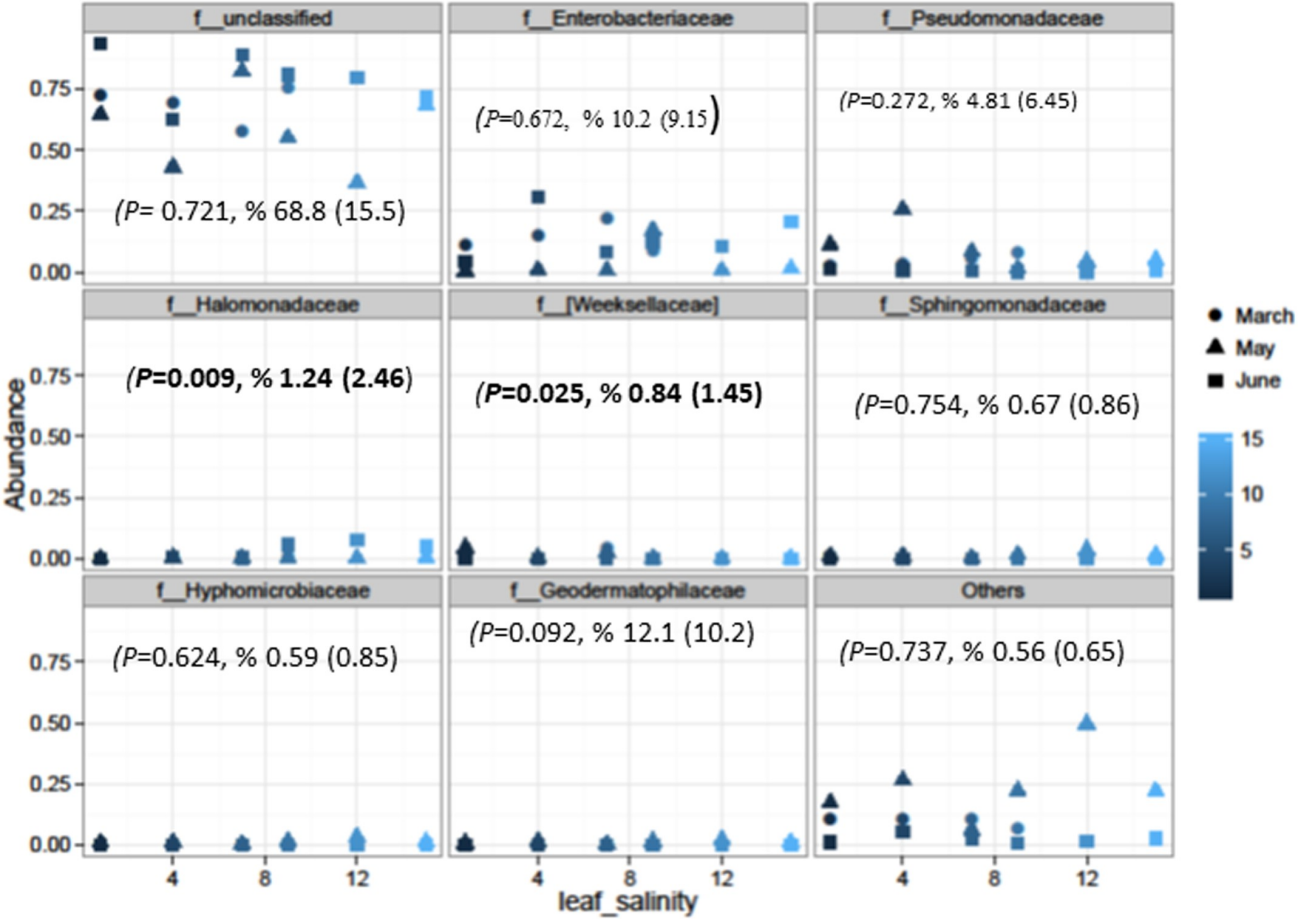
Most Abundant bacteria at the family level in the phyllosphere with no significant differences due to salinity except *Halomonadaceae* and *Weeksellaceae*. Spearman values are shown with relative abundance mean values with standard deviation (SD).

Also, salinity did not affect community composition (PERMANOVA *P* = 0.501; Fig 1) based on the dimensional reduction of the Bray-Curtis distance between microbiome samples, using the PCoA ordination method. Samples were separated by primary axis (Axis 1) and secondary axis (Axis 2) by collection months with March and May samples separating from June samples. March and May samples had more heterogeneity than June samples, as June samples clustered closer to each other. Overall, 67.2% of the sample variation was explained by the major axes. At the family-level abundances *Halomonadaceae* (*P* = 0.0099) and *Weeksellaceae* (*P* = 0.0253) were significantly affected by salinity (Fig 2). There were no significant differences from other families based on Spearman’s test (e.g. *Enterobacteriaceae*: *P* = 0.671; *Pseudomonadaceae*: *P* = 0.272; *Sphingomonadaceae*: *P* = 0.754; *Hyphomicrobiaceae*: *P* = 0.624; Geodermatophilaceae: *P* = 0.737).

Detail analysis was conducted to determine the effect of salinity on bacteria to the species level based on bacterial OTUs. This analysis identified 156 OTUs that were significantly (*P* ≤ 0.05%) impacted by salinity (S1 Table). About 91 OTUs had significant decrease in their F statistics with their log-2fold-change also indicating negative statistics between the first experiment that ended in March and the last experiment that ended in June while 65 OTUs were significantly enriched with positive statistics. Some of these OTUs were unidentified or unclassified, with only thirty-three identified to the genus level and eight to the species level that were significantly enriched (P ≤ 0.05%) due to the impacted of salinity during the study period (Table 3). This data provided additional insight into data presented in Fig 2 with *Halomonadaceae* and *Weeksellaceae* as two of the families that were significantly enriched by salinity. It also provided additional insight into the data that other bacterial species were also significantly affected by salinity during the study. For instance, specific species that were significantly enriched by salinity included *Sphingobacterium multivorum* (0.57-fold, *P*<0.00025) *Acinetobacter Iwoffii* (0.59-fold, *P* < 0.0004), *Methylobacterium adhaesivum* (-0.36-fold, *P* < 0.0059), *Pseudomonas viridiflava* (-0.34-fold, *P* < 0.029), *Candidatus amoebophilu s*(-0.035-fold, *P* < 0.036), *Jeotgalicoccus psychrophilus* (-0.32-fold, *P* < 0.048), *Variovorax paradoxus* (-0.255-fold, *P* < 0.046), *Paenibacillus amylolyticus* (-0.307-fold, *P* < 0.050), and *Bacillus selenatarsenatis*(0.18-fold, *P* < 0.053; Table 3).Generally, salinity might have aided in attenuating species like *Sphingobacterium multivorum*, *Acinetobacter Iwoffii*, *and Bacillus selenatarsenatis* as well as others at the genus level as identified in Table 3 during the study.

**Table 3:** Effect of salinity on bacterial species during the three growing seasons.

Family*	Genus	Species	OTUs	Mean	log2fold-change	lfcSE^$^	stat	pvalue
*Pseudomonadaceae*	*Pseudomonas*	97otu19483	313	6.951	0.502	0.136	3.690	2.24E-04
*Halomonadaceae*	*Halomonas*	unclassified	6	4714.653	0.511	0.139	3.686	2.28E-04
*Sphingobacteriaceae*	*Sphingobacterium*	*multivorum*	723	138.167	0.570	0.156	3.661	2.51E-04
*Moraxellaceae*	*Acinetobacter*	*lwoffii*	373	2.779	0.589	0.166	3.548	3.88E-04
*Cytophagaceae*	*Adhaeribacter*	97otu11598	686	12.113	-0.341	0.097	-3.535	4.07E-04
*Bacteriovoracaceae*	*Bacteriovorax*	97otu97876	1660	5.644	-0.501	0.142	-3.521	4.29E-04
*Piscirickettsiaceae*	*Methylophaga*	97otu41149	279	6.741	0.487	0.147	3.321	8.98E-04
*Bradyrhizobiaceae*	*Balneimonas*	97otu36033	5056	3.687	-0.391	0.121	-3.238	1.20E-03
*Alteromonadaceae*	*Marinimicrobium*	unclassified	1061	2.512	0.522	0.170	3.080	2.07E-03
*Flavobacteriaceae*	*Flavobacterium*	unclassified	9	35.789	0.382	0.125	3.059	2.22E-03
*Cytophagaceae*	*Hymenobacter*	unclassified	352	56.766	-0.423	0.140	-3.029	2.46E-03
*Pseudomonadaceae*	*Pseudomonas*	97otu49044	311	25.868	-0.471	0.167	-2.825	4.73E-03
*Methylobacteriaceae*	*Methylobacterium*	*adhaesivum*	650	57.481	-0.366	0.133	-2.750	5.96E-03
*Pirellulaceae*	*Pirellula*	unclassified	1307	2.555	-0.435	0.164	-2.653	7.97E-03
*Hyphomicrobiaceae*	*Devosia*	97otu501	5334	4.565	-0.333	0.126	-2.632	8.49E-03
*Planctomycetaceae*	*Planctomyces*	97otu15258	524	2.587	0.417	0.166	2.518	1.18E-02
*Microbacteriaceae*	*Agromyces*	unclassified	1065	2.162	0.368	0.152	2.415	1.57E-02
*Weeksellaceae*	*Chryseobacterium*	97otu8996	52	629.246	-0.376	0.157	-2.391	1.68E-02
*Coxiellaceae*	*Aquicella*	97otu72351	1084	2.825	-0.385	0.164	-2.353	1.86E-02
*Weeksellaceae*	*Chryseobacterium*	97otu4579	886	11.357	-0.384	0.164	-2.345	1.90E-02
*Bdellovibrionaceae*	*Bdellovibrio*	97otu51905	2348	4.704	0.333	0.148	2.246	2.47E-02
*Bdellovibrionaceae*	*Bdellovibrio*	unclassified	1716	45.367	-0.213	0.097	-2.197	2.80E-02
*Pseudomonadaceae*	*Pseudomonas*	*viridiflava*	41	879.098	-0.326	0.150	-2.168	3.01E-02
*Amoebophilaceae*	*Candidatus*	*Amoebophilus*	3609	0.829	-0.353	0.165	-2.134	3.29E-02
*Legionellaceae*	*Legionella*	unclassified	4032	0.988	0.354	0.171	2.069	3.86E-02
*Planctomycetaceae*	*Planctomyces*	97otu26717	898	1.028	-0.352	0.171	-2.060	3.94E-02
*Cryomorphaceae*	*Fluviicola*	unclassified	82	4.832	0.275	0.136	2.025	4.28E-02
*Comamonadaceae*	*Variovorax*	*paradoxus*	94	24.823	-0.255	0.128	-1.988	4.68E-02
*Staphylococcaceae*	*Jeotgalicoccus*	*psychrophilus*	2673	1.711	-0.323	0.164	-1.972	4.86E-02
*Bacillaceae*	*Bacillus*	unclassified	5368	28.197	0.187	0.096	1.958	5.02E-02
*Paenibacillaceae*	*Paenibacillus*	*amylolyticus*	129	283.790	-0.307	0.157	-1.953	5.08E-02
*Flavobacteriaceae*	*Flavobacterium*	97otu2979	43	42.293	0.300	0.154	1.951	5.11E-02
*Bacillaceae*	*Bacillus*	*selenatarsenatis*	303	14.307	-0.178	0.092	-1.932	5.33E-02

*Salinity influenced changes in bacterial OTUs at the family level (S1 Table), with 33 identified at the genus level and 8 at the species levels. ^$^lfcSE is the standard error of the log2-Fold-change estimate.

**Table 4:** Wilcoxon signed rank sum test for March and May on top 8 most abundant families affected by season.

Family	P<0.05	March mean (sd)*	May mean (sd)	P<0.05	June mean (sd)	P<0.05	May mean (sd)*	June mean (sd)
*Enterobacteriaceae*	0.20	14.2 (5.85)	4.64 (8.31)	1.00	13.9 (11.9)	0.093	3.38 (6.73)	14.51 (9.74)
*Pseudomonadaceae*	0.36	4.71 (2.49)	11.5 (10.2)	0.10	0.651 (0.452)	0.036	9.2 (8.68)	0.491 (0.433)
*Halomonadaceae*	0.10	0.0174 (0.0285)	0.153 (0.189)	0.10	1.57 (2.79)	0.036	0.169 (0.151)	3.12 (3.35)
*Weeksellaceae*	0.86	1.55 (1.66)	1.77 (2.03)	0.10	0.0421 (0.0762)	0.036	1.19 (1.81)	0.0282 (0.0628)
*Sphingomonadaceae*	0.36	0.588 (0.191)	0.915 (0.508)	0.10	0.031 (0.031)	0.036	1.38 (1.07)	0.036 (0.0274)
*Hyphomicrobiaceae*	0.10	0.304 (0.104)	0.943 (0.441)	0.10	0.0043 (0.0019)	0.036	1.38 (0.98)	0.0082 (0.0063)
*Geodermatophilaceae*	0.10	0.424 (0.0947)	0.983 (0.547)	0.10	0.0232 (0.0157)	0.036	1.21 (0.643)	0.0245 (0.0135)

* Percent relative abundance means are provided. Average temperature in March was 15.9°C, May 17.9°C, June 20.2°C. March is compared to May and June and May is compared to June.

### 3.2. Comparison of leaf microbiomes in response to season

Phyllosphere microbiomes were significantly (*P* = 0.003) affected by season. Air temperatures in experiment 1, 2 and 3 were 15.9°C, 17.9°C, 20.2°C, respectively [22,37]. Leaf surface temperature moderately affect phyllosphere microbiomes (*P* = 0.057), as well as daily mean evapotranspiration (ET0) significantly (*P* < 0.002) affected leaf surface microbiota during the three growing seasons. Daily mean evapotranspiration (ET0) values of each experiment and growing season as number of days was recently reported in a related study [22]. Seasonal water demand during the three experiments vary; ranging in very low in experiment 1, averaging 1.96 mm during the first ten days. During experiments 2 and 3 the minimum ET0 values were 4.43 and 6.36, respectively. It should be noted that the growth period became shorter in the successive seasonal experiments as temperature increased, thus cumulative potential evapotranspiration (PET) decreased as previously reported [22,37] from 276.3, 240.6 and 202.1 mm with increasing temperature for experiment 1, 2 and 3, respectively. High salinity caused yield loss and decreased all gas exchange and vegetative parameters as previously reported [22], and the high salinity may also select for bacteria population with high tolerant to salinity.

**Fig 3:**
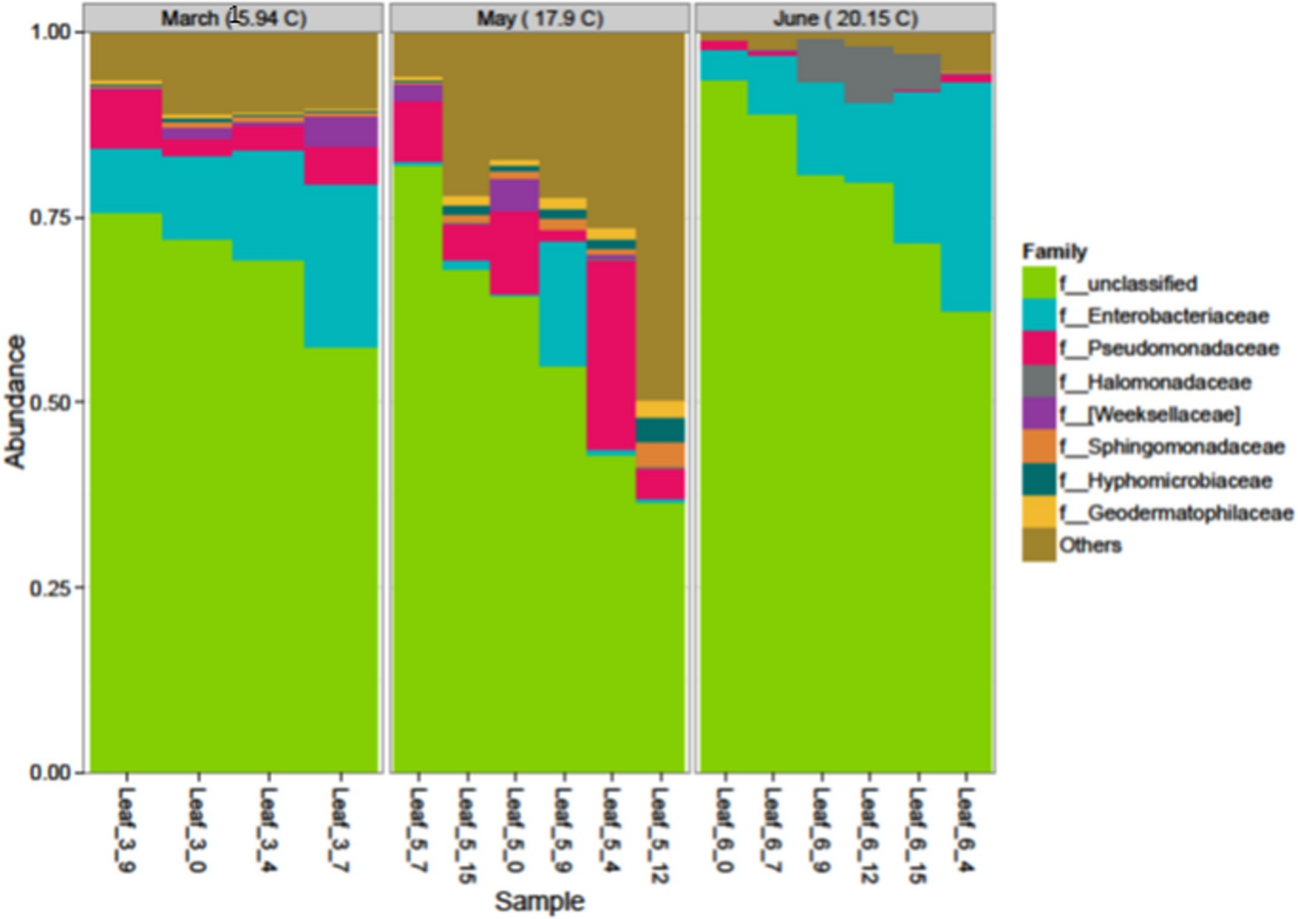
Top 8 most abundant families affected by air temperature during March, May, and June harvesting periods.

The effects of temperature based on season on the most abundant families were analyzed and grouped according to their relative abundances (Fig 3). Wilcoxon signed rank sums and mean relative abundances for family-level taxa between temperature groups (months) are provided in Table 4. The influence of temperature on relative abundance at the family level was not significantly different for all the eight major families between the collection months of March and May based on Wilcoxon signed rank sums and mean relative abundances for family-level taxa (Table 4). No significant effects were also observed between March and June for the eight major taxa. However, seven of the top eight families tested were significantly different between May and June (Table 4).

The effect of season which was associated with increases in temperate showed significant differences (*P* ≤ 0.05%) in the number of OTUs impacted by changes in season due to temperature changes (S2 Table). Very high numbers of OTUs (709) were significantly affected by season in comparison to only 156 that were impacted by salinity. However, only 255 OTUs were impacted negatively by season whereas 454 were significantly enriched. At the genus level, 101 bacteria were identified including 15 identified to the species level suggesting greater effect of season on phyllosphere microbiome than salinity (Table 5). This was also reflected by the high number of log2-folg change as reported in Table 5 in comparison to the smaller changes in salinity. As seen in Table 4 the significant effect of season was observed in six families between the month of May and June harvesting, but none during the first two planting. Our data suggest that more individual bacterial species increased in density than decreased due to season than salinity during the study.

**Table 5:** Effect of season on bacterial species during the three growing seasons.

Family	Genus	Species	OTUs	Mean	*log2fold-change	^$^lfcSE	stat	pvalue
Halomonadaceae	Halomonas	unclassified	6	9187.41	0.483	0.092	5.256	1.47E-07
Methylocystaceae	Pleomorphomonas	97otu48828	1334	36.608	-0.269	0.052	-5.151	2.59E-07
Verrucomicrobiaceae	Prosthecobacter	unclassified	784	15.287	-0.356	0.077	-4.621	3.83E-06
Geodermatophilaceae	Geodermatophilus	unclassified	2023	5.273	-0.451	0.099	-4.546	5.46E-06
Pirellulaceae	Pirellula	97otu96662	5183	4.078	-0.449	0.099	-4.527	5.98E-06
Geodermatophilaceae	Blastococcus	aggregatus	97	29.277	0.512	0.115	4.467	7.93E-06
Opitutaceae	Opitutus	unclassified	229	18.704	-0.298	0.069	-4.295	1.75E-05
Chromatiaceae	Allochromatium	unclassified	50	2529.26	0.495	0.118	4.189	2.80E-05
Peptococcaceae	Desulfosporosinus	meridiei	3261	1.191	-0.409	0.107	-3.834	0.00013
Burkholderiaceae	Burkholderia	unclassified	1713	10.619	0.388	0.101	3.828	0.00013
Nitrospiraceae	Nitrospira	97otu4151	3573	4.489	0.450	0.118	3.822	0.00013
Comamonadaceae	Variovorax	paradoxus	94	14.480	0.314	0.083	3.764	0.00017
Planctomycetaceae	Planctomyces	97otu15258	524	6.099	0.414	0.115	3.595	0.00032
Cytophagaceae	Hymenobacter	97otu85434	795	26.309	0.339	0.094	3.593	0.00033
Leptospiraceae	Leptonema	97otu21598	4658	3.105	-0.378	0.107	-3.528	0.00042
Micromonosporaceae	Catellatospora	97otu7444	3723	4.999	-0.262	0.074	-3.524	0.00043
Xanthomonadaceae	Stenotrophomonas	unclassified	406	4.702	0.358	0.105	3.411	0.00065
Bdellovibrionaceae	Bdellovibrio	97otu87780	6435	1.581	-0.394	0.118	-3.342	0.00083
Idiomarinaceae	Pseudidiomarina	unclassified	796	7.063	0.388	0.117	3.314	0.00092
Nitrososphaeraceae	Candidatus	Nitrososphaera	869	0.852	-0.383	0.116	-3.291	0.00100
Micromonosporaceae	Actinoplanes	97otu54497	1409	4.423	0.415	0.128	3.231	0.00123
Sporichthyaceae	Sporichthya	97otu89295	2632	4.847	0.346	0.111	3.122	0.00180
[Exiguobacteraceae]	Exiguobacterium	97otu8780	631	13.470	-0.246	0.081	-3.050	0.00229
Comamonadaceae	Giesbergeria	97otu59336	293	6.860	-0.183	0.060	-3.046	0.00232
Hyphomicrobiaceae	Pedomicrobium	unclassified	425	36.739	0.322	0.109	2.962	0.00305
Paenibacillaceae	Paenibacillus	amylolyticus	129	42.185	0.333	0.114	2.927	0.00342
Saprospiraceae	Saprospira	unclassified	195	4.387	-0.283	0.097	-2.911	0.00360
Cellulomonadaceae	Cellulomonas	97otu8040	386	14.094	-0.216	0.075	-2.880	0.00397
Nannocystaceae	Nannocystis	exedens	1491	6.724	0.347	0.121	2.868	0.00413
Lachnospiraceae	Dorea	97otu52474	41496	0.782	-0.349	0.123	-2.832	0.00463
Bradyrhizobiaceae	Bradyrhizobium	97otu34922	367	22.279	0.315	0.114	2.769	0.00562
Cytophagaceae	Pontibacter	97otu75711	30943	3.664	-0.192	0.070	-2.766	0.00568
Moraxellaceae	Acinetobacter	guillouiae	66	1.133	0.342	0.126	2.718	0.00656
Pirellulaceae	Pirellula	97otu55909	662	4.743	0.319	0.120	2.656	0.00791
Bacteroidaceae	Bacteroides	fragilis	928	14.350	-0.251	0.096	-2.618	0.00884
Veillonellaceae	Megasphaera	97otu3390	3002	13.340	-0.206	0.079	-2.614	0.00896
Rhabdochlamydiaceae	CandidatusRhabdochlamydia	unclassified	2994	4.643	0.333	0.128	2.598	0.00937
[Pelagicoccaceae]	Pelagicoccus	97otu32654	754	2.535	0.308	0.121	2.545	0.01093
Hyphomicrobiaceae	Hyphomicrobium	97otu1010	224	26.358	0.296	0.117	2.542	0.01103
Xanthomonadaceae	Pseudoxanthomonas	mexicana	99	24.828	-0.213	0.084	-2.538	0.01115
Sphingomonadaceae	Sphingomonas	97otu84915	1389	4.090	0.283	0.114	2.491	0.01276
Bacillaceae	Virgibacillus	picturae	1812	2.074	0.293	0.118	2.479	0.01317
Sphingomonadaceae	Sphingomonas	97otu15062	13284	0.992	-0.289	0.118	-2.440	0.01469
Phyllobacteriaceae	Mesorhizobium	97otu98426	1398	4.208	0.260	0.109	2.395	0.01664
Fimbriimonadaceae	Fimbriimonas	97otu30444	2498	3.932	-0.181	0.076	-2.388	0.01692
Flavobacteriaceae	Flavobacterium	97otu79878	3013	2.251	0.272	0.114	2.388	0.01694
Sphingomonadaceae	Sphingomonas	unclassified	179	50.242	-0.107	0.045	-2.383	0.01716
Nocardiaceae	Rhodococcus	fascians	436	1.320	0.282	0.118	2.379	0.01736
Solibacteraceae	CandidatusSolibacter	unclassified	1143	9.681	0.279	0.118	2.376	0.01750
Planctomycetaceae	Planctomyces	97otu61573	958	7.412	0.217	0.091	2.372	0.01771
Flavobacteriaceae	Flavobacterium	97otu2979	43	38.305	0.149	0.063	2.362	0.01816
Nocardioidaceae	Kribbella	97otu11018	867	9.842	-0.175	0.074	-2.362	0.01817
Cytophagaceae	Adhaeribacter	97otu11598	686	1.313	0.297	0.127	2.349	0.01881
Chitinophagaceae	Segetibacter	97otu13852	2732	2.876	-0.207	0.088	-2.345	0.01903
Hyphomicrobiaceae	Rhodoplanes	97otu11418	638	14.583	0.199	0.085	2.334	0.01960
Caulobacteraceae	Phenylobacterium	97otu70099	899	9.227	0.273	0.118	2.315	0.02061
Hahellaceae	Hahella	97otu62248	1521	3.998	0.231	0.100	2.312	0.02078
Clostridiaceae	Clostridium	97otu74434	2004	9.353	0.274	0.118	2.312	0.02080
Rhodospirillaceae	Magnetospirillum	97otu8709	2601	7.448	0.271	0.118	2.294	0.02180
Euzebyaceae	Euzebya	97otu21482	1190	4.500	0.268	0.118	2.270	0.02318
Paenibacillaceae	Cohnella	97otu5184	17576	0.568	-0.299	0.132	-2.261	0.02375
Gemmataceae	Gemmata	97otu66534	1939	5.467	-0.186	0.084	-2.213	0.02690
Paraprevotellaceae	Prevotella	97otu19469	5823	1.102	0.270	0.122	2.212	0.02697
Cryomorphaceae	Owenweeksia	97otu8278	1903	1.195	0.259	0.117	2.209	0.02719
Rhodobacteraceae	Rubellimicrobium	97otu41659	2572	1.999	-0.252	0.115	-2.198	0.02794
Planctomycetaceae	Planctomyces	97otu26717	898	2.303	0.257	0.117	2.192	0.02839
Planctomycetaceae	Planctomyces	97otu57444	452	25.993	0.170	0.079	2.164	0.03044
Hyphomicrobiaceae	Pedomicrobium	australicum	3098	3.030	0.251	0.117	2.145	0.03192
Fimbriimonadaceae	Fimbriimonas	97otu506	2705	2.626	0.251	0.117	2.138	0.03252
Polyangiaceae	Chondromyces	97otu4353	571	22.886	0.155	0.073	2.133	0.03291
Fimbriimonadaceae	Fimbriimonas	97otu27051	1730	2.702	-0.192	0.090	-2.131	0.03306
Cytophagaceae	Rhodocytophaga	97otu65926	474	0.673	0.282	0.133	2.125	0.03356
Gemmatimonadaceae	Gemmatimonas	97otu11339	538	7.397	0.254	0.120	2.116	0.03435
Planococcaceae	Planomicrobium	unclassified	84365	1.284	0.254	0.120	2.112	0.03466
Lachnospiraceae	Blautia	producta	3267	1.844	0.246	0.117	2.097	0.03602
Micromonosporaceae	Catellatospora	97otu65928	16321	3.047	0.215	0.103	2.094	0.03628
Promicromonosporaceae	Xylanimicrobium	pachnodae	58459	2.247	0.245	0.117	2.092	0.03642
Erysipelotrichaceae	Catenibacterium	97otu5040	327	11.276	0.196	0.094	2.090	0.03660
Ruminococcaceae	Oscillospira	97otu17549	7469	1.918	0.247	0.119	2.087	0.03691
Gemmataceae	Gemmata	97otu55905	3095	1.751	0.247	0.120	2.067	0.03869
Xanthomonadaceae	Lysobacter	97otu22722	2271	0.573	-0.241	0.116	-2.067	0.03871
Cenarchaeaceae	Nitrosopumilus	97otu2230	45	704.189	-0.181	0.088	-2.056	0.03982
Pseudonocardiaceae	Pseudonocardia	unclassified	832	0.925	0.239	0.118	2.033	0.04204
Moraxellaceae	Psychrobacter	pulmonis	3676	0.958	-0.245	0.121	-2.026	0.04273
Planctomycetaceae	Planctomyces	97otu95023	143	25.363	-0.149	0.073	-2.026	0.04276
Enterococcaceae	Enterococcus	unclassified	4462	1.549	0.239	0.118	2.020	0.04342
Gemmataceae	Gemmata	97otu73844	3959	1.642	-0.205	0.102	-2.013	0.04412
Chitinophagaceae	Segetibacter	97otu43467	1055	1.087	0.242	0.121	2.003	0.04514
Intrasporangiaceae	Phycicoccus	97otu67907	177	5.275	0.232	0.116	1.997	0.04585
Hyphomicrobiaceae	Devosia	unclassified	36942	6.045	0.209	0.105	1.995	0.04601
Polyangiaceae	Sorangium	cellulosum	81201	13.730	0.242	0.121	1.992	0.04636
Pirellulaceae	Pirellula	97otu75434	8972	0.972	0.240	0.122	1.973	0.04845
Bacteriovoracaceae	Bacteriovorax	97otu97876	1748	3.216	0.262	0.133	1.964	0.04948
Xanthomonadaceae	Dyella	unclassified	471	1.033	0.263	0.134	1.963	0.04965
Lactobacillaceae	Pediococcus	unclassified	2414	2.502	0.233	0.119	1.958	0.05027
Hyphomicrobiaceae	Rhodoplanes	97otu2443	9043	0.962	0.264	0.135	1.954	0.05068
Sphingobacteriaceae	Sphingobacterium	unclassified	84	63.343	0.196	0.100	1.952	0.05096
Sphingomonadaceae	Kaistobacter	unclassified	73	66.951	-0.118	0.061	-1.940	0.05241
Pseudonocardiaceae	Amycolatopsis	unclassified	78554	0.338	-0.259	0.135	-1.919	0.05498
Cellulomonadaceae	Actinotalea	97otu33606	12437	1.269	0.250	0.131	1.915	0.05551
Bacillaceae	Bacillus	selenatarsenatis	303	1.769	0.208	0.109	1.913	0.05577

*Season influenced changes in bacterial OTUs at the family level (S2 Table), with 101 identified at the genus level and 16 at the species levels. ^$^lfcSE is the standard error of the log2-Fold-change estimate.

In this study salinity significantly affected the family *Halomonadaceae* (*Gammaproteobacteria*) (*P* < 0.009) and *Weeksellaceae* (*P* < 0.025) in the phyllosphere samples throughout the three experiments (Fig 3). *Halomonadaceae* family such as *Halomonas elongata*, is a well-known example of a bacterium that can adapt to life over the whole salt concentration range (halophilic) from near fresh water to halite saturation [38]. The unclassified *Halomonas*, *Sphingobacterium multivorum* and *Acinetobacter Iwoffii* identified in this study had strong positive log2-change, suggesting that these species were not negatively impacted by salinity. In this study, the effects of soil salinity, water content, and how changes in seasons due to warming trends impact bacterial communities on leaf surfaces may provide some guidance into future research in management of saline soils [22,37,39] and changes in leafy green surface microbiome. Therefore, understanding the effect of changes in salinity and water content on soil microorganisms is important for crop production, sustainable land use, and rehabilitation of saline soils [40]. The different ways in which various climate drivers such as temperature, precipitation, ET0 and their interactions with seasons, irrigation methods, and leaf age might affect phyllosphere microorganisms and their activities [9], presents a serious challenge in understanding the impact of drought and salinity on leaf surface bacterial communities. Our study agrees with the work of William et al. [9] emphasizing the difficulties in predicting with full certainty the effects of soil salinity and seasons on leafy green bacterial communities until we collect more long-term data from both control and field studies. As seen from our previous work, the impact of drought/temperature and salinity on bacterial communities or changes in climate drivers may be mostly associated with the rhizosphere [37]. Furthermore, it is expected that the southwestern USA will experience increases in temperature, receive less springtime precipitation, and have more frequent and severe droughts [15]. Overall, climate-induced trends will impact the persistence and dispersal of foodborne pathogens in myriad ways, especially for environmentally ubiquitous microorganisms [14]. Drought can result in decreased bacterial diversity [41], proliferation of pathogenic species [42], and increased survival of an introduced *E*. *coli* O157:H7 derivative [43,44]. Along these lines, drought may inhibit some populations of native microflora while allowing for some of the more resistant groups of pathogenic bacteria, such as *Staphylococcus*, *Clostridium*, and *Bacillus* to survive [14]. These authors noted that most foodborne pathogens are likely to be negatively affected by drought due to their dependence on water activity for survival and growth. Climatic factors such as temperature and season [45–47] may play an important role in shaping community structure.

### 3.3. Population of potential pathogenic bacterial sequences on leaf surfaces

Using Greengenes database (http://greengenes.lbl.gov) to identify potential pathogenic bacteria sequences at the genus level (Table 6), we identify sequences belonging to 18 potential human pathogens and two plant pathogens (Erwinia and Rastonia) among others. These two plants pathogens were included because they have been shown to degrade leaf materials and release carbon sources for pathogens such *E*. *coli* O157 to use and proliferate on leaf surfaces [48]. The relative abundance of the 20-genus identified during the three seasons showed, *Bacillus*, *Pseudomonas*, *Flavobacterium*, *Halomonas*, and *Erwinia* as indicated in Table 6, at the highest abundance levels. Both *Pseudomonas* and *Erwinia* occurred at the highest concentrations in May while *Bacillus* in June. However, most of the sequences associated with potential human pathogens such as *Clostridium*, *Mycobacteria*, *Legionella*, *Nocardia*, *Burkholderia*, *Corynebacterium*, and *Enterococcus* were least abundant in June than in March and May.

**Table 6:** List of some potential human and plant pathogenic bacterial sequence tags identified in spinach phyllosphere in March, May, and June.

Genus	March	May	June*
***Arcobacter***	10	13	16
***Bacillus***	1113	2370	3231
***Burkholderia***	116	58	0
***Clostridium***	35	46	6
***Corynebacterium***	43	187	36
***Enterococcus***	192	83	12
***Erwinia***	1764	63961	35684
***Flavobacterium***	3308	6016	632
***Halomonas***	557	4290	100347
***Lactobacillus***	178	182	52
***Lactococcus***	10	0	0
***Legionella***	3	11	0
***Mycobacterium***	589	1653	59
***Nocardia***	111	340	19
***Nocardioides***	692	1295	9
***Pseudomonas***	115313	260259	15792
***Ralstonia***	170	78	1
***Rhodobacter***	155	371	20
***Rhodococcus***	1241	378	1043
***Serratia***	193	23	139394

*March, May, and June were the months that we collect samples.

*Halomonas* sequences were detected in higher numbers following a warming trend from March to June (Table 6). *Halomonas* is a newly recognized human pathogen causing infections and contamination in a dialysis center [49] and can grow in a wide range of salt concentrations [38]. The detection of sequences for *Arcobacter* and *Acinetobacter* were also of increasing magnitude from March to June (Table 6). Furthermore, *A*. *butzleri* displays microbiological and clinical features like those of *Campylobacter jejuni*; however, *A*. *butzleri* is more frequently associated with watery diarrhea [50]. *Acinetobacter* may also be ubiquitous in the environment and this pathogen is a serious infectious agent of importance to hospitals worldwide [51]. It also can accumulate diverse mechanisms of resistance, leading to the emergence of strains that are resistant to many antibiotics [52]. It should be noted that some of these are human or plant pathogens but many are rather beneficial commensals.

The phyllosphere of spinach during the study was dominated by *Halomonas*, *Pleomorphomonas*, *Prosthecobacter*, *Geodermatophilus*, *Pirellula*, *Blastococcus*, *Opitutus*, *Allochromatium*, *Desulfosporosinus*, *Burkholderiaas*, etc., as affected by temperature. Other studies have shown that lettuce and spinach phyllosphere may contain *Acinetobacter*, *Bacillus*, *Citrobacter*, *Curtobacterium*, *Enterobacter*, *Erwinia*, *Frigoribacterium*, *Methylobacterium*, *Pantoea*, *Pseudomonas*, and *Sphingomonas* as dominant genera [47,53]. It should be noted that bacteria establishing colonies at the phyllosphere are limited by various factors including both biotic and abiotic factors. Abiotic factors such as the available nutrient [1], seasonal variation, rainfall, temperature, plant immunity, and competitor microbes [54] may influence the survival of microbes in the phyllosphere. As previously reported, environmental factors can drastically influence the microbiome changes on phyllosphere. This is common to epiphytic microorganisms exposed to heavy stress during the seasonal cycle, the day/night cycle, and the growth, age, and anatomical dynamics of the plant. As previously reported drought condition can influence the epiphytic microbial community on Holm oak [55]. At the same time, during high temperature, bacterial endophytic communities are altered in lower leaves of paddy, but not in the epiphytes [56], which was in contrast to epiphytic fungal community that responded well during warmer seasons [44,57,58]. Pathogens survival in host tissues can use hemibiotrophs and necrotrophs mode of life. Some chemicals of plant tissues inhibit the microbial association either through salicylic acid or jasmonic acid type and the reactive O_2_ species may have an inhibitory effect on the pathogens [59]. Plants use jasmonic acid, methyl jasmonate, ethylene, flavonoid, 12-oxo-phytodienoic acid, and salicylic acid-mediated signals for quenching pathogens on its surface. Bacteria need carbon, nitrogen, inorganic, and organic energy sources. However, the phyllosphere is a very harsh environment, and in the absence of some of these nutrients, the phyllosphere is still colonized by a large number of bacteria (10^5^–10^7^ CFU/g of the leaf) in the presence of high relative humidity and free water at suitable environmental conditions[60,61]. This is due to the release of nutrients or leaf exudates which adequately can support microbial growth. There are varieties of molecules leached from the plant leaves such as sugar, amino acids, organic acids, minerals, etc. that will also support bacterial growth [62–64]. These leaching materials may differ with plant species and the environmental condition [62,63,65]. Therefore, it is not surprising that in a study like ours, we were still able to identify more than 5000 OTUs using next generation sequencing, and this is in agreement with reports that on the average the phyllosphere may contain large amount of bacteria (10^5^–10^7^ CFU/g of the leaf) [61,62].

### 3.4. Pathways and genes of phyllosphere community

Shotgun sequencing is the direct method for the analysis of functional capabilities of environmental microbiome. However, changes in metabolic pathways and functional genes based on Piphillin algorithm predictions [21] to generate inferred metagenomes of sample type communities based on a 97% identity cutoff has recently been introduced [21]. Approximately 25% of the sample type microbial communities were assigned to genomes by the Piphillin algorithm. This is very low, however other studies have captured the same low correlation between the distance metrics based on phylogenetic/taxonomic and predicted the metagenomic composition of their samples [66,67].

**Fig 4:**
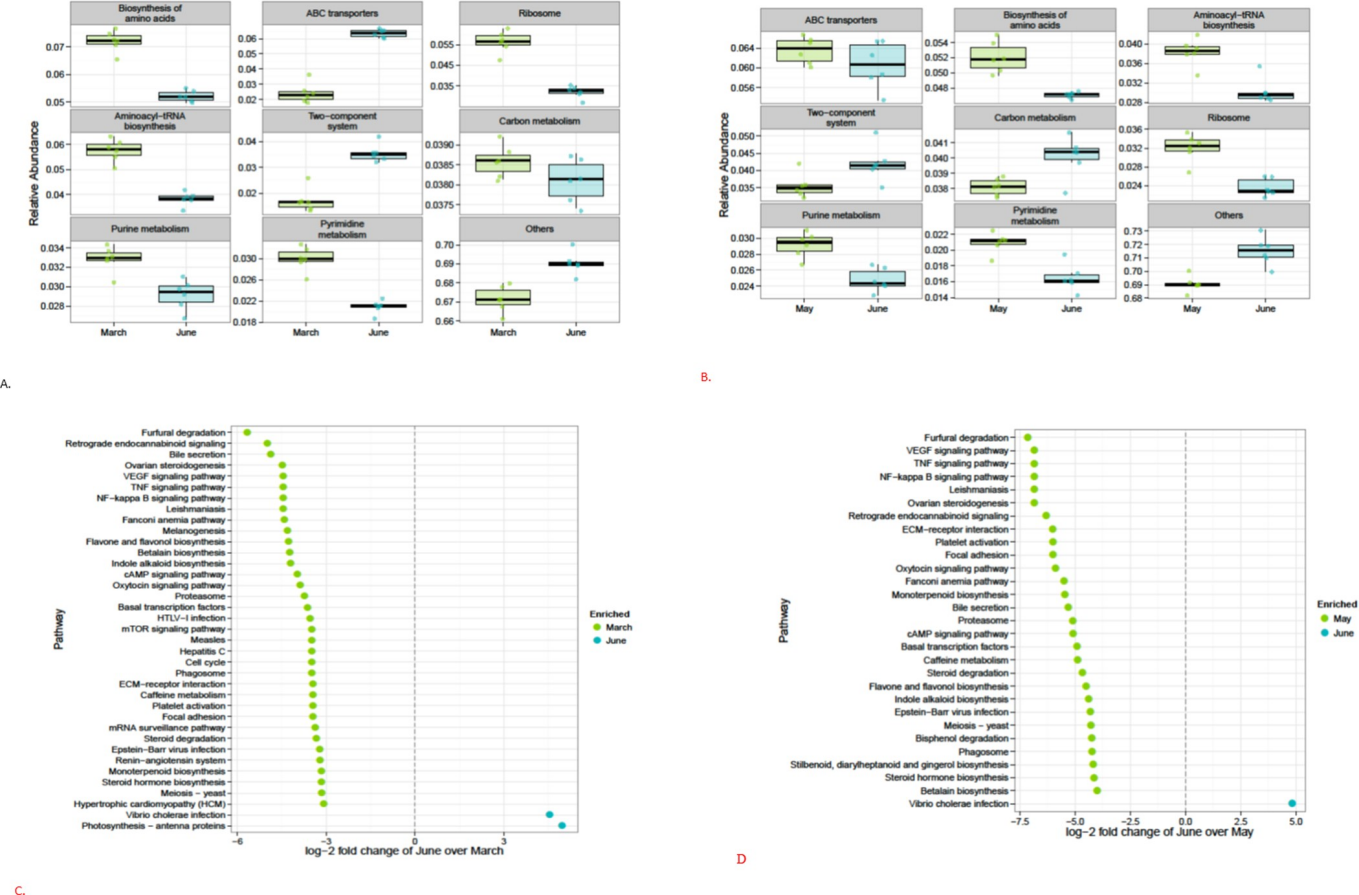
Proportional abundance of the top inferred pathways for March vs. June leaf (Fig 4A); May vs. June (Fig 4B). KEGG pathway prediction feature selection for March vs. June leaf (Fig 4C). There were 161 significantly different features detected out of 308 tested. Only the 37 pathways with an absolute log-2-fold change greater than 3 and not equal to Inf or -Inf are shown. KEGG pathway prediction feature selection for May vs. June leaf (Fig 4D). Features were considered significant if their FDR-corrected P-value was less than or equal to 0.05, and the absolute value of the log-2-fold change was greater than or equal to 1. There were 121 significantly different features detected out of 308 tested. Only the 29 pathways with an absolute log-2-fold change greater than 4 are shown.

The top pathways on leaf samples during the three growing seasons were located to biosynthesis of amino acids, ABC transporters, ribosome, aminoacyl-tRNA biosynthesis, two-component system, carbon metabolism, purine metabolism, and pyrimidine metabolism (Fig 4A and 4B). There were 161 significantly different functional pathway features detected out of 308 tested. Only the 37 functional pathways with an absolute log-2-fold change greater than 3 and not equal to Inf or -Inf are shown. The photosynthesis antenna proteins pathway was significantly enriched in June leaf samples when compared to March (Fig 4C) based on KEGG pathway functional prediction feature selection. Features selections were considered significant if their FDR-corrected P-value was less than or equal to 0.05, and the absolute value of the log-2-fold change was greater than or equal to 1. A total of 157 pathways were enriched in March leaf samples and 4 pathways were enriched in June leaf samples, while 121 pathways were significantly different between the May and June leaf samples. Two pathways were enriched in June samples and 119 pathways were enriched in May samples (Fig 4D). Only the 29 pathways with an absolute log-2-fold change greater than 4 are shown.

**Fig 5:**
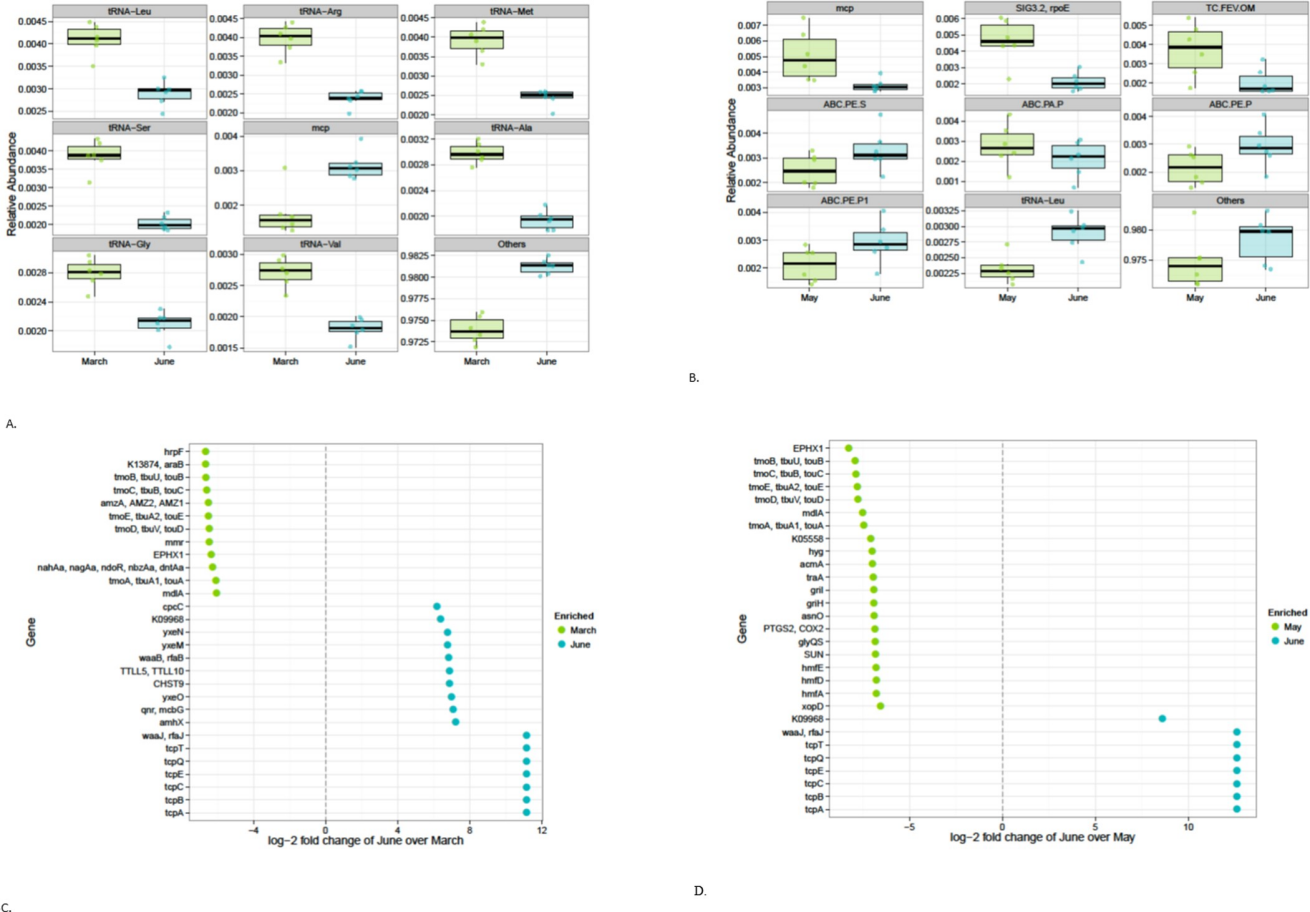
Proportional Abundance of the Top Inferred Genes for March vs. June leaf (Fig 5A). Plot shows the most abundant functional genes. Proportional abundance of the top inferred genes for May vs. June leaf (Fig 5B). Plot shows the most abundant functional genes. KEGG ortholog prediction feature selection for March vs. June leaf (Fig 5C). There were 3,497 significantly different features detected out of 7,127 tested. Only genes with an absolute log-2-fold change greater than 6 and not equal to Inf or -Inf are shown. KEGG ortholog prediction feature selection for May vs. June leaf (Fig 5D). There were 3,033 significantly different features detected out of 7,132 tested. Only 29 genes with an absolute log-2 fold change greater than 6.5 and not equal to Inf or -Inf are shown. Features were considered significant if their FDR-corrected p-value was less than or equal to 0.05, and the absolute value of the log-2 fold change was greater than or equal to 1.

At the gene level, the most abundant genes for March-Junes leaf samples were mcp (methyl-accepting chemotaxis protein), tRNAs genes which included tRNA-alanine, tRNA-arginine, tRNA-glycine, tRNA-leucine, tRNA-methionine, tRNA-serine, and tRNA-valine (Fig 5A), while methyl-accepting chemotaxis protein (mcp), RNA polymerase sigma-70 factor (ECF subfamily; SIG3.2, Sig3.2, rpoE), iron complex outer membrane receptor protein (TC.FEV.OM), polar amino acid transport system permease protein (ABC.PA.P), multiple subunits of the peptide/nickel transport system permease protein (ABC.PE.P1;), and tRNA Leucine were associated with the most abundant genes in May versus June (Fig 5B). There were 3,497 KEGG ortholog significantly different functional pathway prediction features for March versus June that were detected out of 7,127 tested (Fig 5C). Overall, 2,697 genes were enriched in March leaf samples and 800 were enriched in June leaf samples. Only genes with an absolute log-2-fold change greater than 6 and not equal to Inf or -Inf are shown. For May versus June leaf samples, there were 3,033 significantly different features detected out of 7,132 tested. A total of 291 genes were enriched in June samples and 2,742 genes were enriched in May samples. Only 29 genes with an absolute log-2-fold change greater than 6.5 and not equal to Inf or -Inf are shown (Fig 5D). Several genes related to toxin co-regulated pilus biosynthesis proteins were also significantly enriched in June leaf samples, when compared to March and May leaf samples. Overall, several pathways and genes were enriched by different bacterial OTUs with increasing salinity. This may be a function of microbial consortia as individual isolate may not perform all the functions from breaking down the main components and their metabolites in the saline environment [68]. In this study, *Halomonas* was significantly detected in higher numbers following warning tread from March to June in the high saline environment. This suggests that the genus was able to degrade nutrients in the environment for growth. According to Wang et al. [69], the genus *Halomonas* was among a group of bacteria that contributed to phenanthrene degradation intermediates through catechol 1, 2-dioxygenase (C120).

There are a lot of flexible metabolic adaptations for microbes in the phyllosphere, and this is why they survive in the harsh microenvironment. Plant releases a lot of compounds for metabolic functions such as carbohydrates, polyols, amino acids, amines, isoprenoids, halogenated compounds, alcohols, water and salts, and these are the main sources of nutrients for phyllosphere microorganisms [70] as well as saline or alkaline pH which generates stress in phyllosphere microbes. Also, phyllosphere microbes can develop multiple mode of adaptation to survive in phyllosphere such as tolerance, antimicrobial, extracellular polysaccharides, phytohormonal, and immunity compounds against a microbial competitor [70] (Trouvelot et al. 2014). The phyllosphere microbiome acts as a vital role for leaf surface environment and their surrounding ecosystem functions [71]. It has been shown that plants release a variety of volatile organic compounds (VOCs) and its precursors on the surface of leaves such as terpenes, monoterpenes, flavones, methanol, methane, and halogenated methane [72], and these could regulate the microorganisms in response to changes in the environment. Many of the phyllosphere microbial communities in this study share the common metabolic properties of the soil and rhizosphere microbes from our previous study [73]. Phyllosphere bacterial communities such as *Halomonas*, *Pleomorphomonas*, *Prosthecobacter*, *Geodermatophilus*, *Pirellula*, *Blastococcus*, *Opitutus*, *Allochromatium*, *Desulfosporosinus*, *Burkholderiaas*, etc., were also identified in soil and rhizosphere. These bacteria also have carbohydrate metabolizing genes involved in utilization of starch, hemicellulose, pectin, and cellulose, rich in humus materials [74–76]. Therefore, there seems to be some form of synergistic activities involving the above and below ground plant microbiomes.

## 4. Conclusion

In this study, seasonal variations significantly affected bacterial composition on leaf surfaces. Seasonal differences were major factor driving most of the sequences associated with potential human pathogens such as *Clostridium*, *Mycobacteria*, *Legionella*, *Nocardia*, *Burkholderia*, *Corynebacterium*, and *Enterococcus* which were less abundant in June than in March and May. Also, photosynthesis antenna proteins pathway was significantly enriched in June leaf samples in comparison to March, and the high enrichment may be associated with stress conditions such as high light, high salinity, elevated temperature, and nutrient limitation. Therefore, the application of functional pathway analysis has provided another evidence of spinach response to different stress conditions examined in this study during the different seasons and salinity.

## Supporting information

S1 Fig Schematic drawing of the outdoor lysimeter system with 24 outdoor tanks, each connected through PVC pipes and an electric pumping system, with 24 underground water reservoirs. The bird’s view insert (left) shows tank area-oriented N/S with four rows of six tanks each in a caged area of 418 m^2^. Vol = filled volume of reservoir.
(TIF)

S2 Fig The average temperatures (°C) from 1992–2013 was acquired from the California Irrigation Management Systems (CIMIS) weather station no. 44 at University of California Riverside. This covers a 20-year period including the period for this study from December 2012 to June 2013.
(TIF)

S1 Table Leaf salinity effects on bacteria. (XLSX)

S2 Table Effect of temperature on bacterial population. (XLSX)
