# Supplementary figures and images for: Influence of seasonal changes and salinity on spinach phyllosphere bacterial functional assemblage
Source: PLoS One. 2021 Jun 1;16(6):e0252242. doi: 10.1371/journal.pone.0252242 (PMC8168849; doi:10.1371/journal.pone.0252242)

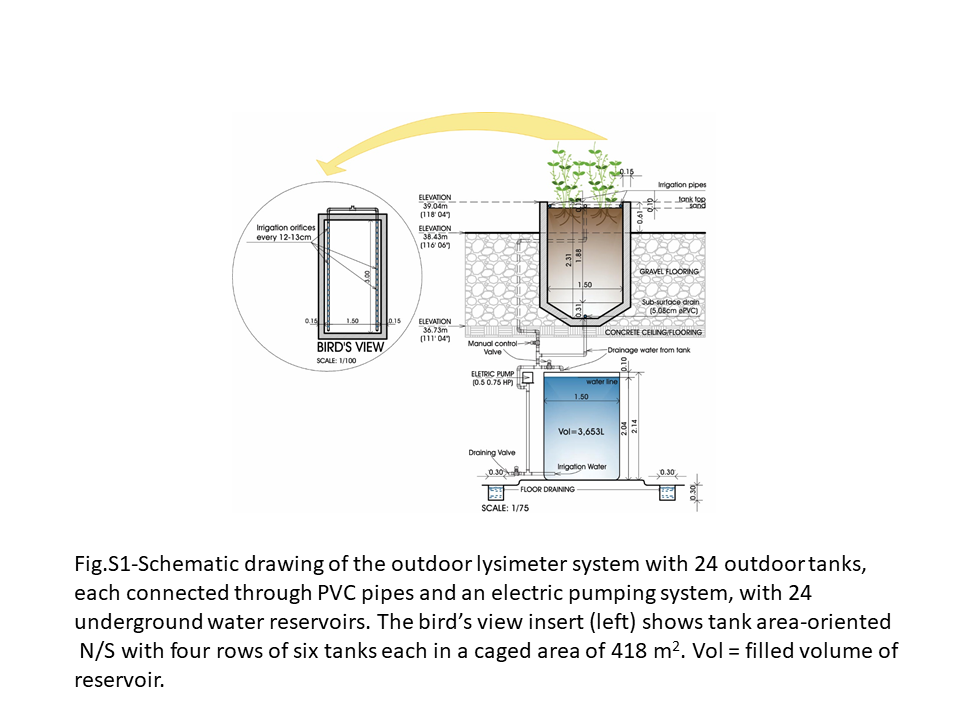

Supplement: S1 Fig — The bird’s view insert (left) shows tank area-oriented N/S with four rows of six tanks each in a caged area of 418 m2. Vol = filled volume of reservoir. (TIF) [file pone.0252242.s001.TIF]

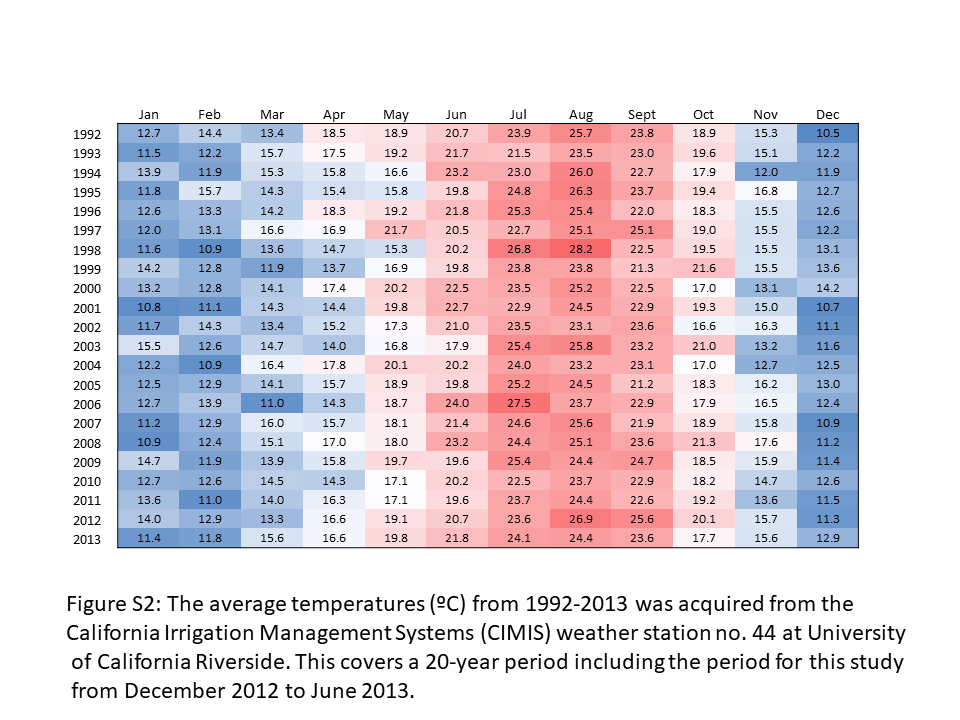

Supplement: S2 Fig — This covers a 20-year period including the period for this study from December 2012 to June 2013. (TIF) [file pone.0252242.s002.TIF]
